# Systemic Immune Modulation in Gliomas: Prognostic Value of Plasma IL-6, YKL-40, and Genetic Variation in YKL-40

**DOI:** 10.3389/fonc.2020.00478

**Published:** 2020-04-17

**Authors:** Camilla Bjørnbak Holst, Ib Jarle Christensen, Jane Skjøth-Rasmussen, Petra Hamerlik, Hans Skovgaard Poulsen, Julia Sidenius Johansen

**Affiliations:** ^1^Department of Radiation Biology, Department of Oncology, Rigshospitalet, Copenhagen University Hospital, Copenhagen, Denmark; ^2^Brain Tumor Biology, Danish Cancer Society Research Center, Danish Cancer Society, Copenhagen, Denmark; ^3^Department of Oncology, Herlev and Gentofte Hospital, Copenhagen University Hospital, Herlev, Denmark; ^4^Department of Medicine, Herlev and Gentofte Hospital, Copenhagen University Hospital, Herlev, Denmark; ^5^Department of Gastroenterology, Hvidovre Hospital, Copenhagen University Hospital, Hvidovre, Denmark; ^6^Department of Neurosurgery, Rigshospitalet, Copenhagen University Hospital, Copenhagen, Denmark; ^7^Department of Clinical Medicine, Faculty of Health and Medical Sciences, University of Copenhagen, Copenhagen, Denmark

**Keywords:** biomarkers, glioblastoma, glioma, IL-6, immune dysfunction, YKL-40

## Abstract

**Background:** Complex local and systemic immune dysfunction in glioblastoma (GBM) may affect survival. Interleukin (IL)-6 and YKL-40 are pleiotropic biomarkers present in the tumor microenvironment and involved in immune regulation. We therefore analyzed plasma IL-6, YKL-40, and genetic variation in YKL-40 and explored their ability to distinguish between glioma subtypes and predict survival in GBM.

**Methods:** One hundred fifty-eight patients with glioma WHO grade II-IV were included in the study. Plasma collected at surgery was analyzed for IL-6 and YKL-40 (CHI3L1) by ELISA. CHI3L1 rs4950928 genotyping was analyzed on whole-blood DNA.

**Results:** Neither plasma IL-6 nor YKL-40 corrected for age or rs4950928 genotype could differentiate GBM from lower grade gliomas. GC and GG rs4950928 genotype were associated with lower plasma YKL-40 levels (CC vs. GC, *p* = 0.0019; CC vs. GG, *p* = 0.01). Only 10 and 14 out of 94 patients with newly diagnosed GBM had elevated IL-6 or YKL-40, respectively. Most patients received corticosteroid treatment at time of blood-sampling. Higher pretreatment plasma IL-6 was associated with short overall survival (OS) [HR = 1.19 (per 2-fold change), *p* = 0.042] in univariate analysis. The effect disappeared in multivariate analysis. rs4950928 genotype did not associate with OS [HR = 1.30, *p* = 0.30]. In recurrent GBM, higher YKL-40 [HR = 2.12 (per 2-fold change), *p* = 0.0005] but not IL-6 [HR = 0.99 (per 2-fold change), *p* = 0.92] were associated with short OS in univariate analysis.

**Conclusion:** In recurrent GBM high plasma YKL-40 may hold promise as a prognostic marker. In newly diagnosed GBM perioperative plasma IL-6, YKL-40, and genetic variation in YKL-40 did not associate with survival. Corticosteroid use may complicate interpretation of results.

## Introduction

In 1863 Rudolf Virchow described a connection between inflammation and cancer (1) and thereby laid the foundation for the present use of immunotherapy as standard of care in several types of cancer. Regrettably, this is not the case for glioblastoma (GBM) yet (2, 3). Despite decades of research, effect of immunotherapy in GBM has been limited (2) and survival remains poor even with intense trimodal standard treatment (4). GBM/CNS (Central nervous system) challenges to immunotherapy include inter- and intratumoral heterogeneity, low tumor mutational burden, local and systemic immune evasion, and restricted drug/immune access to the CNS (2, 3, 5).

The complex brain tumor microenvironment promotes tumor growth, migration, invasion, angiogenesis, and immunosuppression (6–8). Interleukin (IL)-6, a pleiotrophic cytokine, and the chitinase-like glycoprotein YKL-40 are found in glioblastoma tumor cells and cells in the brain tumor microenvironment (7–12), influence tumor propagating mechanisms mentioned above (11–17) and inhibition of YKL-40 or IL-6 decrease tumor growth in xenografted glioma mouse models (15, 17).

IL-6 and YKL-40 regulate each other's transcription levels along with a range of other mediators (9, 18) and IL-6 infusion increased YKL-40 secretion in plasma in healthy volunteers (19). High or increasing levels of circulating IL-6 or YKL-40 have been associated with decreased survival of patients with glioma either alone or in combination with other biomarkers (12, 20–24), although not consistently (25–30) (Supplementary File 1).

The functional *CHI3L1* rs4950928 (-131 C/G) single nucleotide polymorphism (SNP) correlates with plasma YKL-40 levels in a variety of diseases and healthy subjects (31, 32). Exploring rs4950928 SNP as an independent biomarker has revealed ambiguous results (31–34).

The use of circulating biomarkers to assess diagnosis, response to therapy, tumor recurrences, and prognosis for brain tumors has many advantages, including the possibility of repeated measurements and lesser need for invasive surgical procedures. Immune-related plasma biomarker levels reflect systemic immune status, which in GBM may mirror complex neuro-immune interactions and could aid patient- and target-selection for future trials.

Considering the interaction between IL-6 and YKL-40, their potential as prognostic biomarkers and treatment targets in GBM, we explored the prespecified hypotheses that high plasma IL-6 and YKL-40 pre-treatment or at relapse correlate with malignancy grade (WHO grade) of gliomas and have an adverse impact on survival in patients with glioma WHO grade IV. We further investigated whether the *CHI3L1* rs4950928 SNP is related to increased survival through low plasma YKL-40.

## Materials and Methods

### Patients and Patient Samples

**Figure 1:**
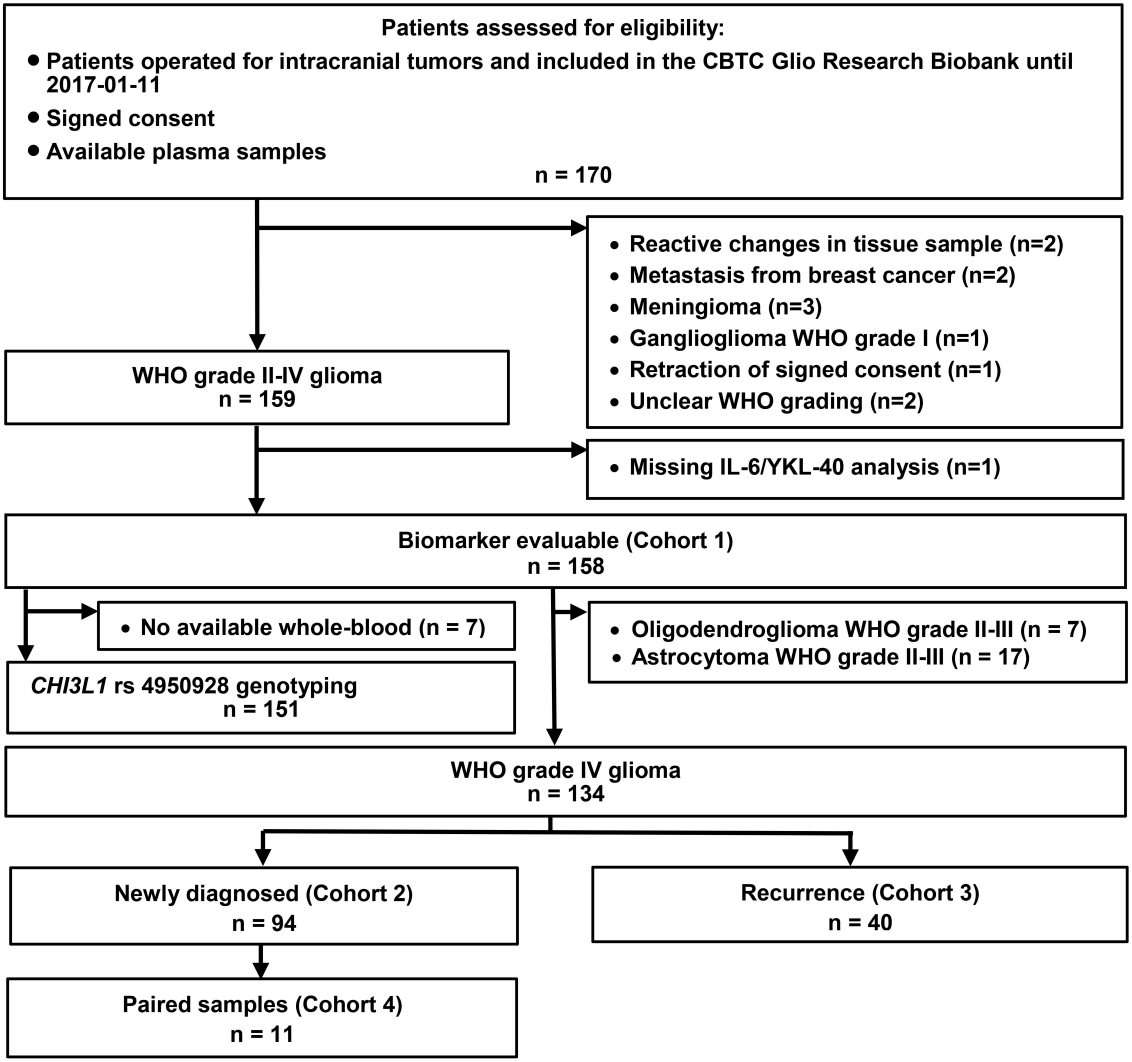
Study populations and patient selection. CBTC, Copenhagen Brain Tumor Consortium.

The Copenhagen Brain Tumor Consortium (CBTC) Glio Research Biobank prospectively includes blood- and tumor samples obtained during surgery from unselected patients with gliomas resected at the Neurosurgical Department, Rigshospitalet, Copenhagen University Hospital, Denmark. All patients included in the Glio Biobank from 2013 until January 2017 with available plasma samples (*n* = 170) were assessed retrospectively for eligibility. Of these, we included 158 biomarker evaluable patients with histologically confirmed WHO grade II-IV gliomas (Figure 1). Sample size was limited by availability of plasma samples. The study cohort includes samples from initial and/or relapse brain tumor surgery (Figure 1). Venous blood samples were collected in EDTA vials (VACUETTE® K2E K2EDTA) at an unspecified time during the surgical procedure and stored at 4°C/on ice for a maximum of 2 h. Plasma was sampled after centrifugation at 3,000 rpm for 10 min at 4°C. Whole-blood and plasma were stored at −80°C until analysis.

The main study cohort (Cohort 1) was further divided into two study populations: Cohort 2 including 94 patients with GBM and blood samples from their initial surgery and Cohort 3 including 40 patients with GBM and blood samples from relapse surgery. Of the 94 patients in Cohort 2, 11 patients with GBM had paired blood samples from initial and relapse surgery (Cohort 4) (Figure 1).

The study was carried out in accordance with the recommendations of the Danish Regional Committee on Health Research Ethics. The protocol was approved by Danish Regional Committee on Health Research Ethics (Region Hovedstaden, H-3-2009-136). All subjects gave written informed consent in accordance with the Declaration of Helsinki.

### Covariates, Clinical Follow-Up, and End Points

Tumor and patient characteristics were found through retrospective review of medical charts, pathology reports, and MRI (magnetic resonance imaging) descriptions. Patients were followed until death or end of follow-up (14th of May 2018).

WHO diagnosis, MGMT (O[6]-methylguanine-DNA methyltransferase) promotor methylation status, 1p-19q chromosomal deletions, ATRX (α thalassemia/mental retardation syndrome X-linked) and IDH (isocitrate dehydrogenase) mutational status were retrieved from pathology reports. Detection methods differed depending on time of analysis. IDH mutations were mostly evaluated by immunohistochemistry (IHC) or by Multiplex Ligation-dependent Probe Amplification (MLPA). MGMT promoter methylation was from 2014 primarily analyzed by pyrosequencing using the Therascreen MGMT Pyro kit (Qiagen) considering a mean methylation above 10% as positive. Until then it was determined indirectly by IHC. Date of progression was found retrospectively through patient charts and MRI descriptions and based on clinician's assessment.

### YKL-40 and IL-6 Plasma Analysis

Plasma concentration of IL-6 and YKL-40 were determined retrospectively at Departments of Medicine and Oncology, Herlev and Gentofte University Hospital, Copenhagen University Hospital, Denmark. Plasma IL-6 levels were determined using an enzyme-linked immunosorbent assay (ELISA) (Quantikine high sensitive IL-6, R&D Systems, Abingdon, Oxon, UK) with a detection limit of 0.01 pg/ml, an intra-assay CV of <10.5%, and inter-assay CV <17.7%, with lowest inter-assay CV (<9%) at high IL-6 concentrations (35). Plasma YKL-40 levels were determined using an ELISA (Quidel, San Diego, California, USA) with a detection limit of 10 ng/ml, an intra-assay coefficient of variation (CV) of <5%, and inter-assay CV <6% (36). For both assays, analyses were performed in accordance with the manufacturers' instructions. The biomedical laboratory scientists were blinded to clinical data. Reference intervals for plasma YKL-40 and IL-6 in healthy subjects have been defined in previous studies with a median plasma YKL-40 concentration of 40 ng/mL (36) and median plasma IL-6 of 1.3 pg/ml, with no significant difference between serum and plasma values (35).

### rs4950928 Genotyping

We retrospectively isolated DNA from whole blood following standard protocol (937255, Qiasymphony, Qiagen). *CHI3L1* rs4950928 genotyping was conducted with Taq-man (Applied Biosystems by Life Technologies Corporation, Carlsbad, CA, USA) assays containing a forward (AGTTCCCATAAAAGGGCTGGTTT) and reverse (CCCAGGCCCTGTACTTCCTTTATAT) primer for the PCR amplification and a common (CTCCCC**C**ACGCGGC) and variant (ACTCCCC**G**ACGCGGC) probe to determine genotype.

### Endpoints and Statistical Analysis

Progression-free-survival (PFS) was calculated for Cohort 2 (newly diagnosed GBM) and defined as time from initial GBM diagnosis (same date as blood-sampling) until first relapse with radiological or clinical progression or death without prior history of relapse. Overall survival (OS) was calculated as time from blood-sampling until date of death of any cause or end of follow-up. Two patients in Cohort 2 were lost to follow-up after first recurrence and were censored for OS analysis.

Plasma concentrations of YKL-40 were converted to age-corrected percentiles using the established normal YKL-40 level in healthy individuals (36) or kept as ng/ml values. All YKL-40 levels referred to are ng/ml unless explicitly called “age-corrected”. Comparisons were made between normal (≤ 95th percentile) and elevated (>95th percentile) (35, 36) IL-6 or age-corrected plasma YKL-40 levels or log_2_ transformed (to ensure linearity) plasma IL-6 and YKL-40. Covariates considered for multivariate analysis are listed in Supplementary File 2.

Association between plasma IL-6 and YKL-40 levels were described using the rank correlation. Fisher's exact test estimated relation between the *CHI3L1* rs4950928 genotype and WHO tumor grade. A general linear model was used to evaluate association between plasma biomarker levels and categorical variables.

Associations between plasma IL-6 and YKL-40 levels and PFS or OS, respectively, were tested using the Cox proportional hazards model estimating univariate and multivariate-adjusted hazard ratios (HR) and 95% confidence intervals (CI). *P* < 5% were considered significant. Multivariate analysis was only made for continuous log_2_ transformed values, since few patients had elevated IL-6 and/or YKL-40 based on pre-specified cut-points. The proportional hazards assumption and linearity were evaluated with martingale residuals. Kaplan-Meier methodology and the log rank test were used for time-to-event endpoints with dichotomized covariates.

Calculations were performed using SPSS (v22.0, IBM Corp., Armonk, NY) or SAS (v9.4, Cary, N.C.USA).

## Results

### Patient Characteristics

Ninety-four (Cohort 2) of 134 patients with GBM were newly diagnosed and 40 patients (Cohort 3) had recurrent GBM at time of blood-sampling (Figure 1). Twenty-four patients presented with WHO grade II-III glioma, nine of these were included in the biobank at relapse surgery. Four out of the 24 WHO grade II-III gliomas were IDH wildtype, three of these later progressed to GBM, whereas the fourth was not resected at relapse. All 7 Oligodendroglioma WHO grade II-III tumors had 1p-19q codeletion analyzed in present or previous tumor samples. Patient characteristics are listed in Supplementary File 3. At end of follow-up 116 patients were dead and 40 patients were alive (2 were lost to follow-up) with a median follow-up time of 134 weeks (Kaplan-Meier method). Median OS in Cohort 2 (newly diagnosed GBM) was 58 weeks (Supplementary File 3).

### Plasma IL-6 and YKL-40 Levels and WHO Grade, GBM Recurrence and Fluctuation Over Time

Plasma IL-6 and YKL-40 levels were elevated in GBM compared to astrocytoma WHO grade II-III (AII-III) patients (*p* = 0.036; *p* = 0.0003). Only plasma YKL-40 levels were higher in patients with GBM (*p* = 0.008) compared to oligodendrogliomas WHO grade II-III (OII-III). When age was added to the model, neither plasma IL-6 or YKL-40 could differentiate GBM from gliomas of lower grade at a given age (IL-6, GBM vs. OII-III *p* = 0.81, IL-6, GBM vs. AII-III *p* = 0.88; YKL-40, GBM vs. OII-III *p* = 0.058; YKL-40, GBM vs. AII-III *p* = 0.29). Exploring the TCGA dataset [http://gliovis.bioinfo.cnio.es/ (37)] *IL6* and *CHI3L1* RNA expression in glioma WHO grade II-IV tumor tissue were both separately able to differentiate IDH wildtype from IDH mutated gliomas (*p* < 0.001; pair-wise *t*-tests with Bonferroni correction), with highest expression in IDH wildtype, which should mainly represent GBMs (Supplementary File 4).

Plasma IL-6 and YKL-40 levels did not differ between the cohort of newly diagnosed and recurrent GBM (IL-6, *p* = 0.49; YKL-40, *p* = 0.066;) (Cohort 2 and 3). Paired plasma samples from 11 patients with GBM (Cohort 4) displayed multi-directional variation in IL-6 and YKL-40 levels between time of diagnosis and relapse (Supplementary File 4). Eight of 11 patients had received standard chemo-radiation therapy [Stupp's regimen (38)] between first and second blood-sampling and none of the eleven patients had MGMT promotor methylation.

### Association Between the *CHI3L1* rs4950928 Genotype, WHO Tumor Grade, YKL-40 Plasma Level, and Survival

**Figure 2:**
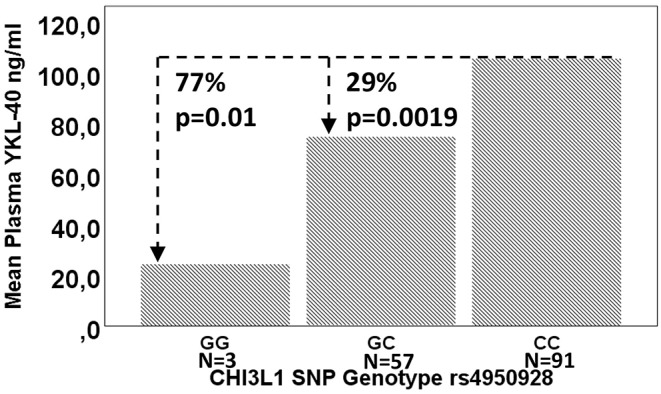
Mean plasma YKL-40 by *CHI3L1* rs4950928 genotype and change in percent. *P*-values are calculated using a general linear model.

For 7 patients, whole-blood was not available for genotype analysis (Figure 1, Supplementary File 3). rs4950928 SNP genotype distribution did not differ between tumor grades [*p* = 0.29 (Fisher's exact test)] but the rs4950928 SNP was significantly associated with lower YKL-40 plasma levels (CC vs. GC, *p* = 0.0019; CC vs. GG, *p* = 0.01) (Figure 2) in Cohort 1. Only 3 patients were homozygote for the GG genotype. Comparing GC with CC genotype there were no significant difference in neither PFS [HR 1.21 (95% CI: 0.76–1.94), *p* = 0.42] or OS [HR 1.30 (95% CI: 0.79–2.12), *p* = 0.3] in newly diagnosed GBM (Cohort 2) in univariate analysis and no impact on OS in recurrent GBM (Kaplan-Meier, *p* = 0.26).

### Associations Between Plasma IL-6 and YKL-40 and Overall Survival in Patients With Newly Diagnosed Glioblastoma

**Figure 3:**
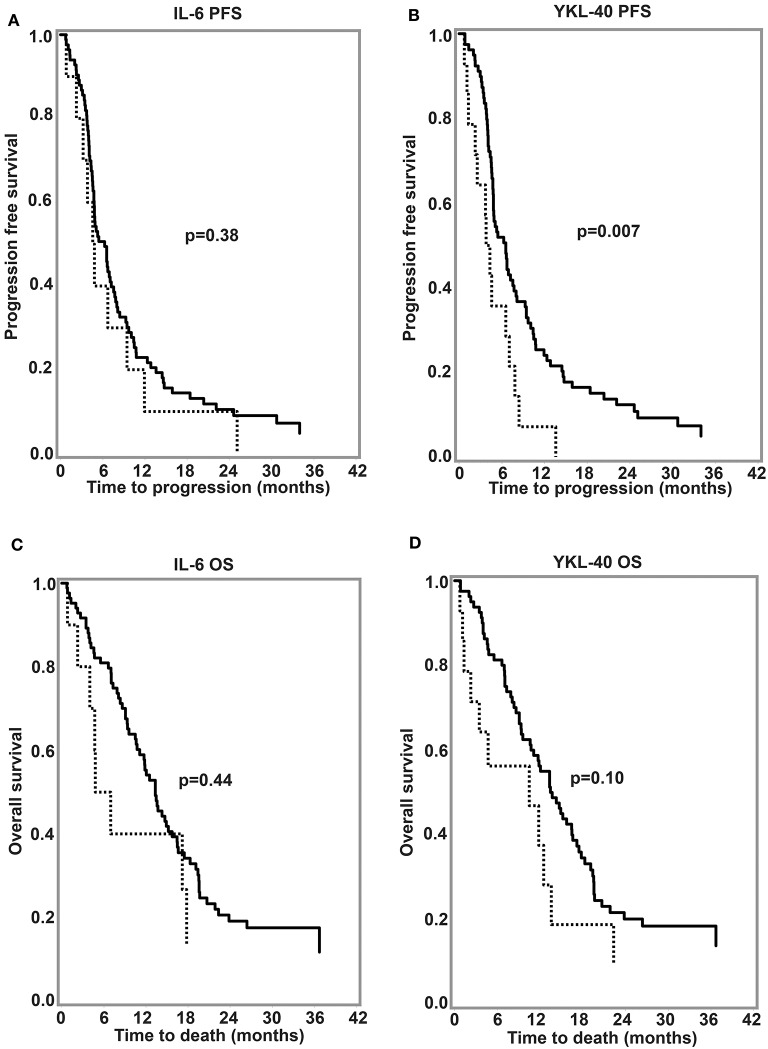
Kaplan-Meier survival curves for **(A,B)** progression-free survival and **(C,D)** overall survival in newly diagnosed glioblastoma according to pretreatment plasma IL-6 and YKL-40. Cut-off: 95th percentile for normal healthy historical controls (4.5 ng/l for IL-6; age-corrected for YKL-40). Dotted lines, >95% percentile; Black lines, ≤95% percentile. *P*-values are calculated using log-rank test. OS, overall survival; PFS, Progression-free survival.

In univariate analysis based on log_2_ transformed plasma biomarker values higher (per 2-fold change) IL-6 concentrations were associated with short OS (HR = 1.19, *p* = 0.042) but not with PFS (HR = 1.14, *p* = 0.084). High plasma YKL-40 was associated with short PFS (HR = 1.27, *p* = 0.011) but not with short OS (HR = 1.19, *p* = 0.070). When divided in high and low plasma IL-6 (cut-off 95% CI in healthy adults; i.e., ≤4.5 ng/l vs. >4.5 ng/l) and YKL-40 (i.e., age-corrected 95% CI for healthy adults), only 10 (10.6%) out of 94 patients had elevated IL-6 and 14 (14.9%) had elevated YKL-40. High age-corrected YKL-40 was associated with short PFS (log-rank *p* = 0.007). Other dichotomizations of biomarker plasma levels did not reveal any association with survival (Figure 3). Analyzing plasma IL-6 and YKL-40 levels against covariate categories in newly diagnosed GBM, GG genotype, and absence of multifocal disease were associated with lower YKL-40 levels, absence of comorbidity was associated with lower IL-6, and increasing age was associated with higher IL-6 and YKL-40 (Table 1). In multivariate analysis neither continuous plasma IL-6 or YKL-40 levels were associated with OS or PFS, whereas gender, treatment and MGMT methylation were independent prognosticators of OS (Table 2) (Reduced model in Supplementary File 5). Multivariate analysis was only made for continuous log_2_ transformed plasma biomarker values considering the small size of groups resulting from the pre-specified dichotomization.

**Table 1:** Pretreatment plasma IL-6 and YKL-40 and patient characteristics for newly diagnosed glioblastoma.

**Plasma biomarkers and patient characteristics**	**Plasma IL-6**			**Plasma YKL-40**		
	**Ratio^a^**	**95% CI**	***p*-value^b^**	**Ratio^a^**	**95% CI**	***p*-value^b^**
rs4950928 Genotype GC vs. CC	1.19	0.80–1.78	0.38	0.76	0.54–1.09	0.13
rs4950928 Genotype GG vs. CC	2.31	0.65–8.20	0.19	0.27	0.09–0.81	0.021
MGMT Met vs. Non-met	1.15	0.77–1.71	0.49	0.75	0.53–1.06	0.099
Age (years) Per 10 years	1.22	1.02–1.45	0.030	1.29	1.11–1.50	0.0011
Gender F vs. M	1.28	0.86–1.89	0.22	0.82	0.58–1.16	0.27
Treatment None/RT/TMZ vs. Stupp^c^	1.43	0.94–2.17	0.095	1.43	0.98–2.07	0.061
Modified CCI^d^ 0 vs. ≥1	0.63	0.41–0.96	0.031	0.70	0.48–1.02	0.066
Multifocal disease No vs. Yes	1.25	0.58–2.66	0.56	0.48	0.24–0.92	0.028
Degree of resection Gross-total vs. Partial^e^	1.09	0.72–1.64	0.68	1.00	0.70–1.43	0.99

a Ratio of plasma biomarker level between categories for each variable;

b A general linear model was used to evaluate association between plasma biomarker levels and categorical variables;

c Treatment regimens were dichotomized into no treatment, radiotherapy only and temozolomide only vs. Stupp's regimen (radiotherapy, concomitant and adjuvant temozolomide) and radiotherapy with concomitant temozolomide;

d Items solid tumor, dementia, and hemiplegia were excluded from the Charlson-Comorbidity index if associated with the brain tumor;

e *Resection status was defined per surgeon's evaluation*.

**Table 2:** Pretreatment plasma IL-6 and YKL-40 and prognosis in newly diagnosed glioblastoma—Multivariate analysis, Full model.

**Multivariate analysis** **Full Model** ***n* = 74**	**PFS**			**OS**		
	**HR**	**95% CI**	***p*-value^a^**	**HR**	**95% CI**	***p*-value^a^**
IL-6 Log_2_ Per 2-fold change	0.97	0.79–1.20	0.80	1.06	0.84–1.33	0.62
YKL-40 Log_2_ Per 2-fold change	1.13	0.89–1.44	0.32	0.99	0.76–1.29	0.96
rs4950928 Genotype GC vs. CC	1.33	0.73–2.43	0.35	1.37	0.72–2.64	0.34
rs4950928 Genotype GG vs. CC	1.71	0.17–16.68	0.65	3.36	0.31–35.97	0.32
MGMT Met vs. Non-met	0.62	0.35–1.09	0.10	0.45	0.24–0.84	0.013
Age (years) Per 10 years	1.02	0.71–1.46	0.91	1.21	0.80–1.84	0.38
Gender F vs. M	1.33	0.73–2.41	0.35	2.08	1.08–4.00	0.028
Treatment None/RT/TMZ vs. Stupp^b^	5.19	2.19–12.32	0.0002	2.98	1.27–7.00	0.012
Modified CCI^c^ 0 vs. ≥1	0.73	0.40–1.35	0.31	0.97	0.50–1.88	0.92
Multifocal disease No vs. Yes	0.52	0.16–1.72	0.29	0.58	0.17–1.96	0.38
Degree of resection Gross-total vs. Partial^d^	0.73	0.41–1.30	0.29	0.75	0.40–1.42	0.38

a Cox regression analysis;

b Treatment regimens were dichotomized into no treatment, radiotherapy only and temozolomide only vs. Stupp's regimen (radiotherapy, concomitant and adjuvant temozolomide) and radiotherapy with concomitant temozolomide;

c Items solid tumor, dementia and hemiplegia were excluded from the Charlson-Comorbidity index if associated with the brain tumor;

d *Resection status was defined per surgeon's evaluation*.

### Recurrent Glioblastoma

None of the patients with recurrent GBM had IL-6 above 4.5 ng/l and only 5 patients had elevated age-corrected YKL-40. In univariate analysis, higher YKL-40 was associated with short OS (HR = 2.12, CI 95%: 1.39–3.23, *p* = 0.0005), whereas IL-6 was not associated with OS (HR = 0.99, CI 95%: 0.74–1.31, *p* = 0.92).

## Discussion

Although the CNS was previously considered an immune-privileged site, it is now evident that neuroimmune interactions within the brain and between brain and circulating immune cells and molecules are important both in disease and normal brain function (39). IL-6 and YKL-40 are both involved in complex immune modulatory systems, cancer propagation, and regulate each other's expression (5, 9, 18, 40, 41). In the present study we evaluated the correlation between IL-6 plasma levels, YKL-40 plasma levels, *CHI3L1* rs4950928 SNP genotype, and survival of newly diagnosed and recurrent GBM. We also explored the association between IL-6 and YKL-40 with WHO tumor grade in gliomas WHO grade II-IV, the association between *CHI3L1* rs4950928 SNP genotype and plasma YKL-40 levels and differences in plasma IL-6 and YKL-40 levels between patients with newly diagnosed and recurrent GBM.

Both plasma IL-6 and YKL-40 levels were higher in GBM compared to astrocytoma WHO grade II-III, but this effect disappeared when corrected for age. Each of the two biomarkers have previously been found to increase in serum with tumor grade (12, 42), but these studies did not account for age differences between patients with GBM and lower grade gliomas.

rs4950928 SNP genotype distribution did not differ between GBM WHO grades. There was an association between rs4950928 SNP genotype and plasma YKL-40 levels in the entire cohort but not with PFS or OS in newly diagnosed GBM or OS in recurrent GBM. This is similar to the results from Boisselier and colleagues, finding a trend toward higher *CHI3L1* RNA expression in GBM tumor tissue in patients with C allele vs. G allele, but no association of the rs4950928 SNP with OS (33). The effect on plasma YKL-40 levels may be a result of methylation on *CHI3L1* CpG sites (43) and changed binding affinity to specific transcription factors (32). Considering the low prevalence of the GG genotype [present study (31, 33)], a larger study is necessary to reject the hypothesis that the GG genotype is linked to survival benefit in GBM.

In patients with newly diagnosed GBM a high plasma IL-6 was associated with short OS in univariate analysis. This effect disappeared in multivariate analysis. IL-6 was associated with age, comorbidity and non-significantly with treatment regimen. Diverging results may therefore reflect selection or confounding bias. Previous studies investigating effect of either plasma or serum IL-6 levels on glioma survival mostly involve small cohorts and use a range of cut-offs, sampling times, and methods of detection (Supplementary File 1). Despite these limitations, eight out of 10 studies (12, 23–29, 44–46) with reported survival outcome do not find association of high plasma IL-6 with short survival (Supplementary File 1), supporting our results. A study on cytokine networks including IL-6 in GBM, found a trend toward survival benefit using a combined IL-4/IL-5/IL-6 serum profile, but no benefit with a partial combination (24), suggesting that combined immune-profiles may be related to GBM propagation and survival.

Investigating YKL-40, we only found high plasma YKL-40 to be associated with short OS in recurrent GBM (univariate analysis). Multivariate analysis on larger sample sizes are necessary to confirm this finding. Postsurgical sample collection (1 week to 48 days after surgery) or measuring changes in plasma or serum YKL-40 predominated studies finding an association between high YKL-40 and short survival (21, 22, 26). YKL-40 increases transiently after glioma surgery (20), whereas IL-6 has been shown to decrease (12), making it very important to standardize time of blood-sampling to gain consistent results. Changes in plasma IL-6 and YKL-40 in glioma could be attributed from tumor mediated regulation of systemic immune responses, as previously suggested for IL-6 (10, 47, 48) and leakage through a damaged blood-brain barrier, but inflammatory diseases (32), steroid treatment (49), neutrophilia (50), and surgical trauma (51, 52) may also influence biomarker plasma levels.

In our study only few patients had elevated IL-6 and YKL-40 compared to historical healthy controls indicating that systemic IL-6 and YKL-40 levels may not be key mediators in glioma-associated systemic immune modulation at time of surgery or that individual changes are more important than the absolute plasma concentration. Considering that corticosteroid treatment may decrease plasma IL-6 (53) and YKL-40 (49) levels and that only 2 out of 82 patients (Supplementary File 3) in our cohort of newly diagnosed GBM did not receive corticosteroid treatment at blood-sampling, this may also at least partly explain the general lack of increase in measured plasma biomarkers. Nevertheless, none of the 5 patients, who received <30 mg steroid daily had increased plasma IL-6 or YKL-40 according to prespecified cut-points and duration of corticosteroid treatment was unknown.

Limitations in the current study include retrospective data collection, incomplete information regarding steroid use, lack of longitudinal measurements, and the unspecified time of blood collection during surgery. A standard glioma surgery has a mean duration of 1.5 h at our institution. Therefore, collecting samples during surgery may have some effect on IL-6, but only little effect on plasma YKL-40 levels based on previous IL-6 and TNF-alfa stimulation in healthy individuals (19).

In conclusion, plasma YKL-40 at time of surgery may predict survival in recurrent GBM. Plasma IL-6, YKL-40, and genetic variation in YKL-40 are not associated with survival at initial GBM surgery but may be difficult to interpret in patients treated with corticosteroids. Exploring the potential of more comprehensive combined immune profiles as a minimally invasive simple method of portraying local and systemic immune dysregulation is highly relevant.

## Data Availability Statement

The datasets for this article are not publicly available due to participant confidentiality. Requests to access the datasets should be directed to CH, camilla.bjoernbak.holst@regionh.dk.

## Ethics Statement

The studies involving human participants were reviewed and approved by Danish Regional Committee on Health Research Ethics (Region Hovedstaden, H-3-2009-136). The patients/participants provided their written informed consent to participate in this study.

## Author Contributions

Patient inclusion, sample collection, and preparation were performed by JS-R and PH. Data collection and analysis were performed by CH, IC, and JJ. The first draft of the manuscript was written by CH. Review and editing were performed by all authors. All authors approved the final manuscript and contributed to the study conception and design.

## Conflict of Interest

The authors declare that the research was conducted in the absence of any commercial or financial relationships that could be construed as a potential conflict of interest.
