# Supplementary material for: Systemic Immune Modulation in Gliomas: Prognostic Value of Plasma IL-6, YKL-40, and Genetic Variation in YKL-40
Source: Front Oncol. 2020 Apr 17;10:478. doi: 10.3389/fonc.2020.00478 (PMC7180208; doi:10.3389/fonc.2020.00478)
Supplement: Supplementary file 1 [file Data_Sheet_1.PDF]

Supplementary Material

Supplementary file 1: Studies measuring serum/plasma IL-6 in patients with glioma

Reference	No. of patients	Cut-off	Time of blood-sampling	Survival end-point	Detection	Effect on survival	Covariates in multivariate analysis
Albulescu et al. (2013) [1]	GBM (55)	Did not use cut-off		No survival end-point	xMAP assay, Luminex 200 system, Human cytokine 12-plex kit		
Batchelor et al. (2010) [2]	Recurrent GBM (31)	”Biomarker levels measured on quantitative scales were log-transformed and changes were calculated as ratios of on-study to baseline values”	Before cediranib therapy and 8 hours, 1, 9, 28, 56, 84 and 112 days thereafter	Radiographic progression, Mortality	Multiplex enzyme-linked immunosorbent assay plates (Meso-Scale Discovery, Gaithersburg, MD)	Pretreatment:<br><br>Progression: HR -8; 95% CI -17-2; p=0.12<br><br>Mortality: HR -4; 95% CI -12-5; p=0.36<br><br>Change at 8 hours after cediranib treatment:<br><br>Progression: HR -26; 95% CI -64-52; p=0.40	

						Mortality: HR -27; 95% CI -69-72; p=0.45<br><br>Change at 1 day after cediranib treatment<br><br>Progression: HR -22; 95% CI -51-23; p=0.29<br><br>Mortality: HR -18; 95% CI -29-96; p=0.52	
Batchelor et al. (2013) [3]	GBM(40)	Log-transformed covariates, biomarker changes were expressed as ratios, reported as median with interquartile intervals	”Before and after cediranib-chemoradiation therapy at days 1, 2, and 14, and then weekly until the end of combination therapy (week 10)”	PFS and OS		No association with outcome	
Buneviciu s et al. (2018) [4]	HGG (48), LGG (21)	≥ 2 pg/ml		12-month and 60-month mortality risk<br><br>Assessment of survival: time	Radioimmunoassay method (Roche Cobas analyzer, Roche	HGG 12-month: OR 4.068; 95% CI 1.664-9.946; p=0.002	Patient age (years), gender, tumor histological diagnosis (HGGvsLGG), adjuvant treatment

				in days from hospital discharge date until time of death	Diagnostics, UK)	HGG 60-month: OR 2.623; 95% CI 1.129-5.597; p=0.01	(yes vs. no), extent of resection, elevated hsCRP
Carlsson et al. (2010) [5]	GBM (18)		Pre-operatively and during immunization 4 and 8 (autologous IFN-γ transfected glioma cells)		Human recombinant scFv antibody microarray		
Chiorean et al. (2014) [6]	GBM (14)	Median value (>42 ng/ml)	Before surgical resection	DFS and OS	Commercial ELISA (R&D Systems, Minneapolis, MN)	All patients:<br><br>DFS: HR 1.44; 95% CI 0.43-4.75; p=0.53<br><br>OS: HR 1.36; 95% CI 0.41-4.48; p=0.59	
Demirci et al. (2012) [7]	GBM (38), AA (6)	Median value (>3.73)	2-3 weeks after surgery	PFS and OS	Commercial ELISA (R&D Systems, Minneapolis, MN, USA)	All patients: PFS: p=0.69<br><br>OS: p=0.40<br><br>(Univariate log-rank test)	

Doroudchi et al. (2013) [8]	Glioma (38)	Did not use cut-off	Before surgery and/or before therapy	No survival end-point	Biotin-avidin commercial ELISA assay (BMS213/2, ebioscience, USA)		
Gerstner et al. (2011) [9]	GBM (17)	Continuous log transformed	Prior to therapy, 8h, 1 day, 2 days, 9 days, 50 days and 4 weeks after completion of radiation	PFS and OS	Multiplex ELISA (Meso-Scale Discovery, Gaithersburg, MD)	Not reported	
Kalpathy-Cramer et al. 2017 [10]	Recurrent GBM (10)	Changes were expressed as an absolute difference from baseline measurements	Baseline, within 24-72h after treatment initiation with tivozanib and monthly thereafter	PFS and OS	Human Proinflammatory-4 Kit (K15025A)	No association with OS or PFS<br><br>Change from Cycle 1 day 2 to baseline (Cox Univariate analysis):<br><br>PFS: HR 0.81; 95% CI 0.24-2.67; p=0.73	
Kmiecik et al. (2013) [11]	GBM (10)	-	During surgical resection	Dit not measure survival	Cytometric Bead Assay (BD Biosciences)	Not relevant	-

Nijaguna et al. (2015) [12]	GBM (148)	-	Prior to surgery		ELISA	Not reported	
Reynés et al. (2011) [13]	GBM (40)	3.6 pg/ml	Before surgery	Survival and progression-free survival	Commercial ELISA (High Sensitivity Human IL-6 ELISA kit, Diaclone)	No association between marker level and survival or PFS (Kaplan-Meier, log rank test)	-
Shan et al. (2015) [14]	Glioma WHO I-II (18), Glioma WHO III (25), GBM (43)	>20 ? (serum; before surgery)<br><br>>50% decrement (change from before to one month after surgery)	24 h before surgery and 1 month after surgery	Survival time and progression-free survival	Double-antibody sandwich ELISA (HUYU Biological Technology Co., Ltd., Shanghai)	IL-6 in serum and decrement after surgery were related to prognosis (Kaplan-Meier)	
Xu et al. (2012) [15]	GBM (23, plasma), Glioma III (5),	Median protein expression level	Immediately before surgery	Survival	Luminex multiplex immunoassay	Did not have significant prognostic value (log-rank test)	
Zhenjiang et<br><br>al. (2018) [16]	GBM WHO IV (145), Glioma WHO II-III (60)	'Median concentration values of cytokines in samples which could generate the	Day of surgery, prior to initiation of cancer therapy	Overall survival	Commercial ELISA (MABTECH, Stockholm, Sweden)	Forward and backward stepwise multivariate analysis:<br><br>GBM: IL-4/IL-5/IL-6: OS: HR:	Radiotherapy, Chemotherapy, EBNA-1, Survivin$_{97-111}$, Serum IFN-γ/TNF-α/IL-17A

		greatest hazard ratios between two groups'				1.7851; 95% CI 0.996-3.20; p=0.052	

Supplementary file 1: Studies measuring serum/plasma YKL-40 in patients with glioma

Reference	No. of patients	Cut-off	Time of blood-sampling	Survival end-point	Detection	Effect	Covariates in multivariate analysis
Bernardi et al. (2012) [17]	60	YKL-40 ratio ((1-week value-baseline value)/baseline value) (time-dependent covariate or dichotomization on ≥0.5 or ≥ 1)<br><br>Baseline YKL-40 (time-dependent covariate)<br><br>1st week YKL-40 (time-	Preoperatively (fasting), 1 week, 1 month and every 3 months after surgery	OS (time from the date of surgery to date of death or last follow-up)	Quantitative immunoassay (Quidel Corporation, San Diego, CA)	Univariate Cox analysis on OS:<br><br>Baseline YKL-40: 1.01 (0.99-1.02); p=0.549<br><br>1st week YKL-40: 1.01 (1.003-1.015); p=0.004<br><br>YKL-40 ratio: 2.53 (1.37-4.63); p=0.003<br><br>Multivariate Cox analysis on OS:<br><br>YKL-40 ratio: 1.97 (1.03-3.8); p=0.040	Age, extent of resection

		dependent covariate)					
Boisen et al. (2018) [18]	563 (GBM)<br><br>AVAglio trial	>90th percentile of normal	Within 29 to 48 days after surgery (before treatment with RT/TMZ and bev/placebo), additional samples were taken at regular intervals and at progression	PFS and OS	Commercial ELISA (Quidel, San Diego, California)	All patients:<br><br>PFS: HR 1.84; 95% CI 1.20-2.80; p=0.0047<br><br>OS: HR 1.94; 95% CI 1.23-3.06; p=0.0042<br><br>(Multivariate analysis)	Treatment BEV vs Pl, Age, Race, WHO PS, MGMT, Type of surgery, corticosteroid use at baseline, gender, MMSE score, delay between surgery and subsequent treatment, primary vs secondary glioblastoma, enzyme-inducing anti-epileptic drug use at baseline, confirmation of GBM histology
Gallego Pérez-Larraya et al. (2014) [19]	111 (GBM)	Probably 60 ng/ml	Immediately before surgery	PFS (time from surgery to disease progression or death), OS (date of surgery or last follow-up)	Commercial ELISA (TECOmedical SARL, Versailles, France)	PFS: HR 1.09; 95% CI 0.83-1.42; p=0.54<br><br>OS: HR 1.21; 95% CI 0.89-1.64; p=0.23<br><br>(Univariate analysis)<br><br>Multivariate analysis: YKL-40	Age, KPS, extent of surgery, tumor size, plasma levels of IGFBP-2 and GFAP.

						not included in final model	
Hormigo et al. (2006) [20]	77 (GBM) (75 included in survival analysis); 66 (Glioma III)	Continuous log scale (per doubling of YKL-40 values)<br><br>Change in markers compared with the first level determined for each patient (per doubling of YKL-40 values).	All blood samples were obtained within 4 weeks of MRI, further for some patients undergoing surgery samples were taken within 14 days preoperatively and serially 1-14 days postoperatively	OS (time from registration to date of death or last follow-up)	Commercial ELISA (Quidel, San Diego, California)	GBM:<br><br>Continuous YKL-40 OS: HR 1.4; 95% CI 1.1-1.9; p=0.02<br><br>Change in YKL-40: No association with survival p=0.12<br><br>Anaplastic glioma:<br><br>Continuous YKL-40: no association with survival p=0.26<br><br>Change in YKL-40: OS: HR 1.7; 95% CI 0.94-3.2; p=0.08<br><br>Anaplastic astrocytoma:<br><br>Continuous YKL-40: OS: HR 2.2;	

						95% CI 0.99-4.9; p=0.05<br><br>Change in YKL-40: OS: HR 2.3; 95% CI 0.98-5.2; p=0.06<br><br>(Univariate analysis)	
Iwamoto et al. (2011) [21]	197 (GBM) (165 included in multivariate analysis), 105 (Glioma III) (98 included in multivariate analysis), 41 (LGG)<br><br>58 (newly diagnosed GBM, trial)<br><br>143 of the patients included are also reported in Hormigo et al. 2006	Change in YKL-40 (per doubling of YKL-40 values)<br><br>≥98 ng/ml	Patients could enroll at any time during disease, serum samples were taken at baseline and every 2-3 months<br><br>First blood-sample within 3 months of diagnosis was considered newly-diagnosed	Survival (time from study registration to date of death or last follow-up)	Commercial ELISA (Quidel, San Diego, California)	GBM:<br><br>Change in YKL-40: OS HR 1.4; 95% CI 1.2-1.6; $p<0.0001$<br><br>(Multivariate analysis)<br><br>Newly-diagnosed GBM (subgroup):<br><br>≥98 ng/ml baseline: OS HR 1.2; 95% CI 1.0-1.4; p=0.03<br><br>(Kaplan-Meier)<br><br>Single baseline measurement not prognostic when adjusted for prognostic factors	Age, extent of resection at diagnosis, baseline KPS, tumor burden

						Newly-diagnosed GBM (trial patients)<br><br>Change in YKL-40: OS: HR 1.5; 95% CI 1.1-2.0; p=0.01<br><br>PFS: HR 0.93; 95% CI 0.7-1.2; p=0.51<br><br>Anaplastic glioma:<br><br>Change in YKL-40: OS: HR 1.4; 95% CI 1.1-1.9; p=0.01<br><br>(Multivariate analysis)<br><br>Anaplastic astrocytoma:<br><br>Change in YKL-40: OS: HR 1.5; 95% CI 1.1-2.1; p=0.008<br><br>(Univariate analysis)	

Tanwar et al. (2002) [22]	45 (GBM); 20 (Glioma II-III)	No cut-off		No survival end-point	Commercial ELISA (Metra/Later named Quidel Biosystems)		
van Linde et al. (2016) [23]	47 (GBM)		Postoperative (median 21 days from surgery until sampling), after chemoradiation, after completion of adjuvant treatment phase	PFS (time between surgery and disease progression), OS (time between surgery and death)	ELISA (Sunred Biological Technology Co)	YKL-40 not associated with PFS p=0.54 (Cox regression analysis)	

Studies measuring serum/plasma IL-6 or YKL-40 in patients with glioma. AA, anaplastic astrocytoma; DFS, disease-free survival; GBM, glioblastoma; HGG, high-grade glioma, LGG, low-grade glioma; OS: overall survival; PFS: progression-free survival

References

1. Albulescu R, Codrici E, Popescu ID, Mihai S, Necula LG, Petrescu D, Teodoru M, Tanase CP (2013) Cytokine patterns in brain tumour progression. Mediators of inflammation 2013:979748. doi:10.1155/2013/979748
2. Batchelor TT, Duda DG, di Tomaso E, Ancukiewicz M, Plotkin SR, Gerstner E, Eichler AF, Drappatz J, Hochberg FH, Benner T, Louis DN, Cohen KS, Chea H, Exarhopoulos A, Loeffler JS, Moses MA, Ivy P, Sorensen AG, Wen PY, Jain RK (2010) Phase II study of cediranib, an oral pan-vascular endothelial growth factor receptor tyrosine kinase inhibitor, in patients with recurrent glioblastoma. Journal of clinical oncology : official journal of the American Society of Clinical Oncology 28 (17):2817-2823. doi:10.1200/jco.2009.26.3988
3. Batchelor TT, Gerstner ER, Emblem KE, Duda DG, Kalpathy-Cramer J, Snuderl M, Ancukiewicz M, Polaskova P, Pinho MC, Jennings D, Plotkin SR, Chi AS, Eichler AF, Dietrich J, Hochberg FH, Lu-Emerson C, Iafrate AJ, Ivy SP, Rosen BR, Loeffler JS, Wen PY, Sorensen AG, Jain RK (2013) Improved tumor oxygenation and survival in glioblastoma patients who show increased blood perfusion after cediranib and

chemoradiation. Proceedings of the National Academy of Sciences of the United States of America 110 (47):19059-19064. doi:10.1073/pnas.1318022110
4. Bunevicius A, Radziunas A, Tamasauskas S, Tamasauskas A, Laws ER, Iervasi G, Bunevicius R, Deltuva V (2018) Prognostic role of high sensitivity C-reactive protein and interleukin-6 in glioma and meningioma patients. Journal of neuro-oncology 138 (2):351-358. doi:10.1007/s11060-018-2803-y
5. Carlsson A, Persson O, Ingvarsson J, Widegren B, Salford L, Borrebaeck CA, Wingren C (2010) Plasma proteome profiling reveals biomarker patterns associated with prognosis and therapy selection in glioblastoma multiforme patients. Proteomics Clinical applications 4 (6-7):591-602. doi:10.1002/prca.200900173
6. Chiorean R, Berindan-Neagoe I, Braicu C, Florian IS, Leucuta D, Crisan D, Cernea V (2014) Quantitative expression of serum biomarkers involved in angiogenesis and inflammation, in patients with glioblastoma multiforme: correlations with clinical data. Cancer biomarkers : section A of Disease markers 14 (2-3):185-194. doi:10.3233/cbm-130310
7. Demirci U, Yaman M, Buyukberber S, Coskun U, Baykara M, Uslu K, Ozet A, Benekli M, Bagriacik EU (2012) Prognostic importance of markers for inflammation, angiogenesis and apoptosis in high grade glial tumors during temozolomide and radiotherapy. International immunopharmacology 14 (4):546-549. doi:10.1016/j.intimp.2012.08.007
8. Doroudchi M, Pishe ZG, Malekzadeh M, Golmoghaddam H, Taghipour M, Ghaderi A (2013) Elevated serum IL-17A but not IL-6 in glioma versus meningioma and schwannoma. Asian Pacific journal of cancer prevention : APJCP 14 (9):5225-5230. doi:10.7314/apjcp.2013.14.9.5225
9. Gerstner ER, Eichler AF, Plotkin SR, Drappatz J, Doyle CL, Xu L, Duda DG, Wen PY, Jain RK, Batchelor TT (2011) Phase I trial with biomarker studies of vatalanib (PTK787) in patients with newly diagnosed glioblastoma treated with enzyme inducing anti-epileptic drugs and standard radiation and temozolomide. Journal of neuro-oncology 103 (2):325-332. doi:10.1007/s11060-010-0390-7
10. Kalpathy-Cramer J, Chandra V, Da X, Ou Y, Emblem KE, Muzikansky A, Cai X, Douw L, Evans JG, Dietrich J, Chi AS, Wen PY, Stufflebeam S, Rosen B, Duda DG, Jain RK, Batchelor TT, Gerstner ER (2017) Phase II study of tivozanib, an oral VEGFR inhibitor, in patients with recurrent glioblastoma. Journal of neuro-oncology 131 (3):603-610. doi:10.1007/s11060-016-2332-5
11. Kmiecik J, Poli A, Brons NH, Waha A, Eide GE, Enger PO, Zimmer J, Chekenya M (2013) Elevated CD3+ and CD8+ tumor-infiltrating immune cells correlate with prolonged survival in glioblastoma patients despite integrated immunosuppressive mechanisms in the tumor microenvironment and at the systemic level. Journal of neuroimmunology 264 (1-2):71-83. doi:10.1016/j.jneuroim.2013.08.013
12. Nijaguna MB, Patil V, Hegde AS, Chandramouli BA, Arivazhagan A, Santosh V, Somasundaram K (2015) An Eighteen Serum Cytokine Signature for Discriminating Glioma from Normal Healthy Individuals. PloS one 10 (9):e0137524. doi:10.1371/journal.pone.0137524
13. Reynes G, Vila V, Martin M, Parada A, Fleitas T, Reganon E, Martinez-Sales V (2011) Circulating markers of angiogenesis, inflammation, and coagulation in patients with glioblastoma. Journal of neuro-oncology 102 (1):35-41. doi:10.1007/s11060-010-0290-x
14. Shan Y, He X, Song W, Han D, Niu J, Wang J (2015) Role of IL-6 in the invasiveness and prognosis of glioma. International journal of clinical and experimental medicine 8 (6):9114-9120

15. Xu BJ, An QA, Srinivasa Gowda S, Yan W, Pierce LA, Abel TW, Rush SZ, Cooper MK, Ye F, Shyr Y, Weaver KD, Thompson RC (2012) Identification of blood protein biomarkers that aid in the clinical assessment of patients with malignant glioma. International journal of oncology 40 (6):1995-2003. doi:10.3892/ijo.2012.1355
16. Zhenjiang L, Rao M, Luo X, Valentini D, von Landenberg A, Meng Q, Sinclair G, Hoffmann N, Karbach J, Altmannsberger HM, Jager E, Peredo IH, Dodoo E, Maeurer M (2018) Cytokine Networks and Survivin Peptide-Specific Cellular Immune Responses Predict Improved Survival in Patients With Glioblastoma Multiforme. EBioMedicine 33:49-56. doi:10.1016/j.ebiom.2018.06.014
17. Bernardi D, Padoan A, Ballin A, Sartori M, Manara R, Scienza R, Plebani M, Della Puppa A (2012) Serum YKL-40 following resection for cerebral glioblastoma. Journal of neuro-oncology 107 (2):299-305. doi:10.1007/s11060-011-0762-7
18. Boisen MK, Holst CB, Consalvo N, Chinot OL, Johansen JS (2018) Plasma YKL-40 as a biomarker for bevacizumab efficacy in patients with newly diagnosed glioblastoma in the phase 3 randomized AVAglio trial. Oncotarget 9 (6):6752-6762. doi:10.18632/oncotarget.22886
19. Gallego Perez-Larraya J, Paris S, Idbaih A, Dehais C, Laigle-Donadey F, Navarro S, Capelle L, Mokhtari K, Marie Y, Sanson M, Hoang-Xuan K, Delattre JY, Mallet A (2014) Diagnostic and prognostic value of preoperative combined GFAP, IGFBP-2, and YKL-40 plasma levels in patients with glioblastoma. Cancer 120 (24):3972-3980. doi:10.1002/cncr.28949
20. Hormigo A, Gu B, Karimi S, Riedel E, Panageas KS, Edgar MA, Tanwar MK, Rao JS, Fleisher M, DeAngelis LM, Holland EC (2006) YKL-40 and matrix metalloproteinase-9 as potential serum biomarkers for patients with high-grade gliomas. Clinical cancer research : an official journal of the American Association for Cancer Research 12 (19):5698-5704. doi:10.1158/1078-0432.ccr-06-0181
21. Iwamoto FM, Hottinger AF, Karimi S, Riedel E, Dantis J, Jahdi M, Panageas KS, Lassman AB, Abrey LE, Fleisher M, DeAngelis LM, Holland EC, Hormigo A (2011) Serum YKL-40 is a marker of prognosis and disease status in high-grade gliomas. Neuro-oncology 13 (11):1244-1251. doi:10.1093/neuonc/nor117
22. Tanwar MK, Gilbert MR, Holland EC (2002) Gene expression microarray analysis reveals YKL-40 to be a potential serum marker for malignant character in human glioma. Cancer research 62 (15):4364-4368
23. van Linde ME, van der Mijn JC, Pham TV, Knol JC, Wedekind LE, Hovinga KE, Aliaga ES, Buter J, Jimenez CR, Reijneveld JC, Verheul HM (2016) Evaluation of potential circulating biomarkers for prediction of response to chemoradiation in patients with glioblastoma. Journal of neuro-oncology 129 (2):221-230. doi:10.1007/s11060-016-2178-x
