# Supplementary material for: Systemic Immune Modulation in Gliomas: Prognostic Value of Plasma IL-6, YKL-40, and Genetic Variation in YKL-40
Source: Front Oncol. 2020 Apr 17;10:478. doi: 10.3389/fonc.2020.00478 (PMC7180208; doi:10.3389/fonc.2020.00478)
Supplement: Supplementary file 2 [file Data_Sheet_2.PDF]

Supplementary Material

Supplementary file 2: Covariates

Covariates included in multivariate analysis

- age (per 10-year increase)
- gender (female vs. male)
- modified CCI without age (0 vs. ≥ 1)
- MGMT promotor methylation status (methylated vs. non-methylated)
- multifocal disease (no vs. yes)
- degree of tumor resection (gross-total resection vs. partial resection)
- first line oncological treatment (None/radiation/temozolomide vs. Stupp's regimen/radiation and concomitant temozolomide)

Covariates originally considered, but excluded from statistical analysis

- smoking habits
- alcohol consumption
- performance status (PS)
- steroid intake
- reresection
- treatment regimen at first recurrence

Resection status in cohort 2 was defined per surgeon's evaluation and divided into partial and gross-total resection. Stereotactic biopsies were grouped with partial resections. We modified the Charlson-Comorbidity index (CCI) [1] to exclude items solid tumor, dementia and hemiplegia if associated with the brain tumor. This was to exclude the effect from the brain tumor itself and to avoid influence from fluctuating neurological symptoms. Alcohol consumption, smoking habits and multifocality were only assessed at primary surgery.

Covariates smoking habits, alcohol consumption, performance status (PS) and steroid intake were considered for statistical analysis but were excluded on the grounds of missing data, due to incomplete or imprecise medical charts. Reresection and treatment at first recurrence was not included in multivariate analysis because of ensuing reduction in n, since 19 patients died prior to recurrence or were alive without recurrence at follow-up (Supplementary file 3).

Reference

1. Charlson ME, Pompei P, Ales KL, MacKenzie CR (1987) A new method of classifying prognostic comorbidity in longitudinal studies: development and validation. Journal of chronic diseases 40 (5):373-383. doi:10.1016/0021-9681(87)90171-8
