# Supplementary material for: Systemic Immune Modulation in Gliomas: Prognostic Value of Plasma IL-6, YKL-40, and Genetic Variation in YKL-40
Source: Front Oncol. 2020 Apr 17;10:478. doi: 10.3389/fonc.2020.00478 (PMC7180208; doi:10.3389/fonc.2020.00478)
Supplement: Supplementary file 3 [file Data_Sheet_3.pdf]

Supplementary Material

Supplementary file 3: Patient Characteristics

**Characteristics (Cohort 1)**	**Gliomas WHO grade II-IV (n=158)**
**Age, years median (range)**	60 (23-89)
**Gender, n (%)**	
Male	94 (59.5)
Female	64 (40.5)
**Diagnosis, n (%)**	**n=158**
Oligodendroglioma WHO grade II-III	7 (4.4%)
Astrocytoma WHO grade II-III	17 (10.8%)
GBM	134 (84.8%)
**Survival outcome**	**n=156**
OS, weeks median (range)	58 (1-233)
Lost to follow-up	2
**Plasma YKL-40 ng/ml median (range)**	62 (10-1035)
**Plasma IL-6 pg/ml median (range)**	1 (0.3-26.5)
***CHI3L1* SNP rs4950928, n (%)**	**n=151**
C/C	91 (60.3)
G/C	57 (37.7)
G/G	3 (2.0)
Missing	7
**Status at end of follow-up, n (%)**	**n=156**
Dead	116 (74.4)
Alive	40 (25.6)
Lost to follow-up	2

**Characteristics (Cohort 2)**	**Newly diagnosed GBM (n=94)**
**Age, years median (range)**	65 (33-89)
**Gender, n (%)**	
Male	57 (60.6)
Female	37 (39.4)
**Modified Charlson Comorbidity Index, n (%)**	**n=89**
0	62 (69.7)
1	19 (21.3)
2	4 (4.5)
3	2 (2.2)
4	0
5	0
6	2 (2.2)
Missing	5
**Multifocal disease, n (%)**	**n=85**
Yes	6 (7.1)
No	79 (92.9)

Missing	9
**Degree of resection, n (%)**	**n=87**
Partial resection	41 (47.1)
Gross-total resection	46 (52.9)
Missing	7
**Treatment, n (%)**	**n=94**
No treatment	7 (7.4)
Radiation therapy only	3 (3.2)
Temozolomide (TMZ) only	2 (2.1)
Stupps Regimen	65 (69.1)
Radiation therapy and concomittant TMZ	2 (2.1)
Palliative radiation therapy	15 (16)
Missing	0
**Steroid treatment at blood-sampling, n (%)**	**n=82**
0	2 (2.4)
12.5-25 mg	3 (3.7)
50-75 mg	28 (34.1)
87.5-100 mg	46 (56.1)
>100 mg	3 (3.7)
Missing	12
**MGMT promotor methylation status, n (%)**	**n=91**
Methylated	40 (44)
Non-methylated	51 (56)
Missing	3
**Recurrence, n (%)**	**n=93**
Yes	74 (77.7)
Dead without verified recurrence	13
Alive at end of follow-up without recurrence	6
Missing (Uncertainty regarding recurrence date)	1
**Survival outcome**	
PFS, weeks median (range)	23 (3-233)
Missing (Uncertainty regarding recurrence date), n	1
OS, weeks median (range)	58 (3-233)
Lost to follow-up, n	2
**Plasma YKL-40 ng/ml median (range)**	77.5 (10-1035)
**Plasma IL-6 pg/ml median (range)**	1.1 (0.3-26.5)
***CHI3L1* SNP rs4950928, n (%)**	**n=90**
C/C	59 (65.6)
G/C	29 (32.2)
G/G	2 (2.2)
Missing	4

**Characteristics (Cohort 3)**	**Recurrent GBM (n=40)**
**Age, years median (range)**	56 (37-79)
**Gender, n (%)**	

Male	22 (55)
Female	18 (45)
**Modified Charlson Comorbidity Index, n (%)**	**n=39**
0	31 (79.5)
1	3 (7.7)
2	4 (10.3)
3	1 (2.6)
Missing	1
**Multifocal disease, n (%)**	**n=38**
Yes	4 (10.5)
No	34 (89.5)
Missing	2
**Primary treatment regimen, n= 38 (%)**	**n=38**
No treatment	1 (2.6)
Radiation therapy only	0
Temozolomide (TMZ) only	0
Stupps Regimen	35 (92.1)
Radiation therapy and concomittant TMZ	0
Palliative radiation therapy	2 (5.3)
Missing	2
**Number of recurrences, n (%)**	**n=39**
First recurrence GBM	25 (64.1)
Second recurrence GBM	12 (30.8)
Third recurrence GBM	2 (5.1)
Missing	1
**Treatment, n (%)**	**n=38**
No treatment	18 (47.4)
Radiation therapy only	1 (2.6)
Temozolomide only	6 (15.8)
Lomustine only	1 (2.6)
Avastin and Lomustine	5 (13.2)
Clinical trial	5 (13.2)
Radiation therapy and Temozolomide	2 (5.2)
Missing	2
**Survival outcome**	
OS (from blood sampling to death or end of follow-up), weeks median (range)	33 (2-102)
OS (from diagnosis to death or end of follow-up), weeks median (range)	97 (30-785)
**Plasma YKL-40 ng/ml, median (range)**	60.5 (17-266)
**Plasma IL-6 pg/ml, median (range)**	1.1 (0.3-4.4)
***CHI3L1* SNP rs4950928, n (%)**	**n=37**
C/C	20 (54.1)
G/C	17 (45.9)
G/G	0
Missing	3
