# Supplementary material for: Systemic Immune Modulation in Gliomas: Prognostic Value of Plasma IL-6, YKL-40, and Genetic Variation in YKL-40
Source: Front Oncol. 2020 Apr 17;10:478. doi: 10.3389/fonc.2020.00478 (PMC7180208; doi:10.3389/fonc.2020.00478)

Supplementary Material

Supplementary file 4
IL6 and CHI3L1 RNA expression (A) in glioma WHO grade II-IV tumor tissue from the TCGA dataset, downloaded from http://gliovis.bioinfo.cnio.es/ (Bowman et al., 2017). (B) Pretreatment plasma IL-6 and YKL-40 in patients with newly diagnosed glioblastoma (Cohort 2) versus recurrent glioblastoma (Cohort 3). (C) Plasma IL-6 and YKL-40 in paired samples from initial GBM surgery and at first recurrence (Cohort 4). GBM, glioblastoma; IDHmut-codel, IDH mutated 1p-19q codeleted; IDHmut-non-codel, IDH mutated 1p-19q non codeleted; IDHwt, IDH wildtype

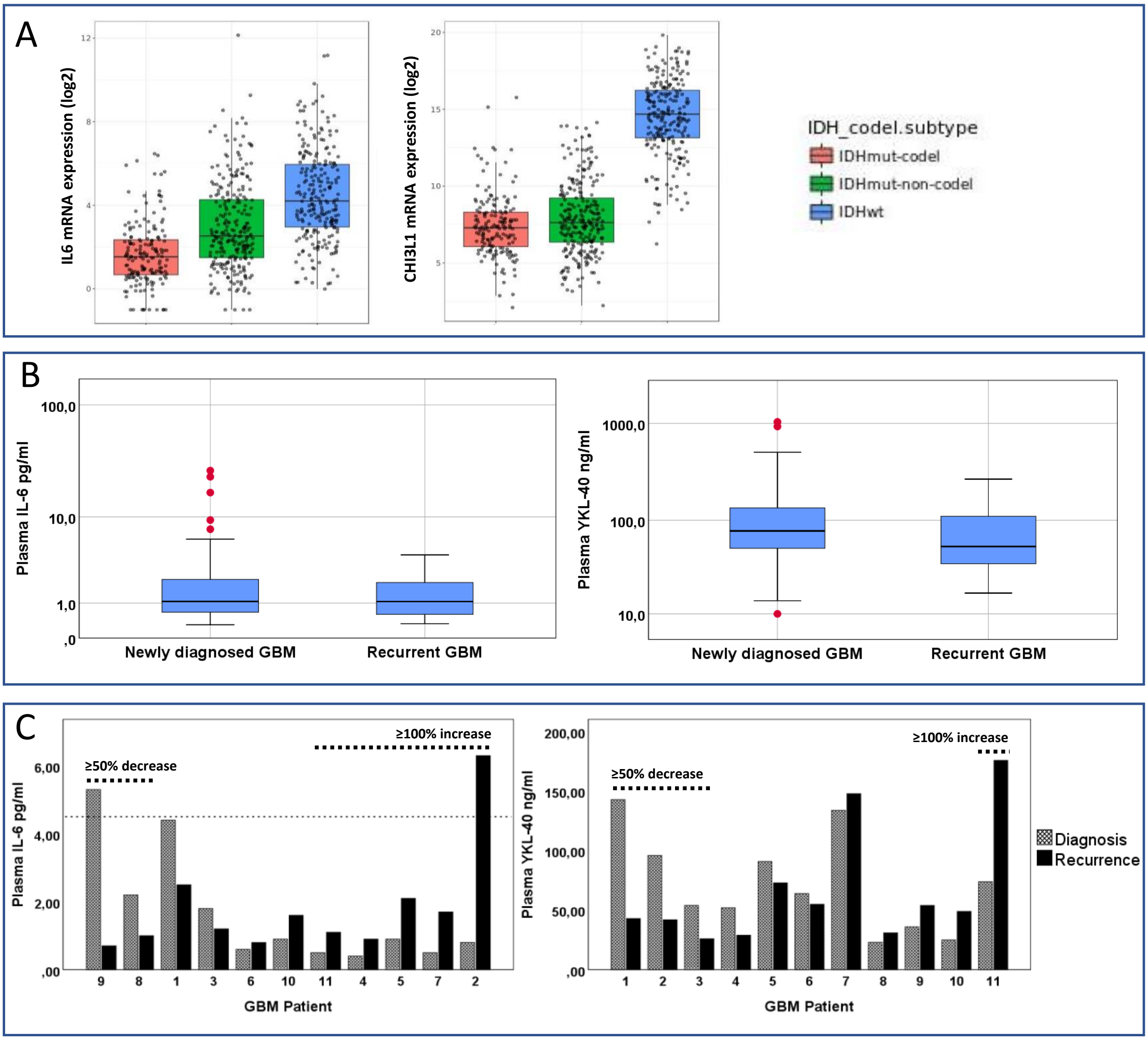

Supplement: Supplementary file 4 [file Data_Sheet_4.pdf]
