# Supplementary material for: Systemic Immune Modulation in Gliomas: Prognostic Value of Plasma IL-6, YKL-40, and Genetic Variation in YKL-40
Source: Front Oncol. 2020 Apr 17;10:478. doi: 10.3389/fonc.2020.00478 (PMC7180208; doi:10.3389/fonc.2020.00478)
Supplement: Supplementary file 5 [file Data_Sheet_5.pdf]

Supplementary Material

Supplementary file 5: Multivariate analysis, Newly diagnosed GBM, Reduced model

**Multivariate analysis Reduced Model n=87**	**PFS**			**OS**		
	HR	95% CI	p-value[a]	HR	95% CI	p-value[a]
**IL-6 Log_2 Per 2-fold change**	0.98	0.82-1.16	0.79	1.03	0.86-1.24	0.76
**YKL-40 Log_2 Per 2-fold change**	1.22	0.99-1.49	0.058	1.09	0.89-1.33	0.39
**MGMT Met vs Non-met**	0.57	0.35-0.93	0.024	0.48	0.28-0.83	0.008
**Age (years) Pr 10 years**	0.95	0.72-1.25	0.71	1.05	0.78-1.41	0.76
**Gender F vs M**	1.30	0.80-2.13	0.29	1.92	1.14-3.23	0.014
**Treatment None/RT/TMZ vs Stupp[b]**	6.60	3.21-13.56	<0.0001	4.04	2.01-8.13	<0.0001

[a] Cox regression analysis; [b] Treatment regimens were dichotomized into no treatment, radiotherapy only and temozolomide only versus Stupp's regimen (radiotherapy, concomitant and adjuvant temozolomide) and radiotherapy with concomitant temozolomide

A reduced model for multivariate analysis was developed, since 20 patients were excluded from the full model because of missing data (Table 2, Supplementary file 3). Apart from IL-6 and YKL-40, covariates were included in reduced analysis if they had a statistically significant effect on PFS or OS in full multivariate analysis. YKL-40 is closely associated to age, therefore age was also included.
